# Ecological and public health dimensions of ESBL-producing *Escherichia coli* in bats: A One Health perspective

**DOI:** 10.14202/vetworld.2025.1199-1213

**Published:** 2025-05-17

**Authors:** Alfiana Laili Dwi Agustin, Aswin Rafif Khairullah, Mustofa Helmi Effendi, Wiwiek Tyasningsih, Ikechukwu Benjamin Moses, Budiastuti Budiastuti, Hani Plumeriastuti, Sheila Marty Yanestria, Katty Hendriana Priscilia Riwu, Fidi Nur Aini Eka Puji Dameanti, Wasito Wasito, Riza Zainuddin Ahmad, Agus Widodo, Daniah Ashri Afnani

**Affiliations:** 1 Doctoral Program of Veterinary Science, Faculty of Veterinary Medicine, Universitas Airlangga, Jl. Dr. Ir. H. Soekarno, Kampus C Mulyorejo, Surabaya, 60115, East Java, Indonesia; 2 Department of Veterinary Public Health, Faculty of Veterinary Medicine, Universitas Pendidikan Mandalika, Jl. Pemuda No. 59A, Dasan Agung Baru, Mataram, 83125, West Nusa Tenggara, Indonesia; 3 Research Center for Animal Husbandry, National Research and Innovation Agency (BRIN), Jl. Raya Bogor Km. 46 Cibinong, Bogor, 16911, West Java, Indonesia; 4 Department of Veterinary Public Health, Faculty of Veterinary Medicine, Universitas Airlangga, Jl. Dr. Ir. H. Soekarno, Kampus C Mulyorejo, Surabaya, 60115, East Java, Indonesia; 5 Department of Veterinary Microbiology, Faculty of Veterinary Medicine, Universitas Airlangga, Jl. Dr. Ir. H. Soekarno, Kampus C Mulyorejo, Surabaya, 60115, East Java, Indonesia; 6 Department of Applied Microbiology, Faculty of Science, Ebonyi State University, Abakaliki, 480211, Nigeria; 7 Study Program of Pharmacy Science, Faculty of Health Science, Universitas Muhammadiyah Surabaya, Jl. Raya Sutorejo No.59, Dukuh Sutorejo, Mulyorejo, Surabaya, 60113, East Java, Indonesia; 8 Department of Veterinary Pathology, Faculty of Veterinary Medicine, Universitas Airlangga, Jl. Dr. Ir. H. Soekarno, Kampus C Mulyorejo, Surabaya, 60115, East Java, Indonesia; 9 Department of Veterinary Public Health, Faculty of Veterinary Medicine, Universitas Wijaya Kusuma Surabaya, Jl. Dukuh Kupang XXV No.54, Dukuh Kupang, Dukuh Pakis, Surabaya, 60225, East Java, Indonesia; 10 Laboratory of Veterinary Microbiology and Immunology, Faculty of Veterinary Medicine, Universitas Brawijaya, Jl. Puncak Dieng, Kunci, Kalisongo, Dau, Malang, 65151, East Java, Indonesia; 11 Department of Health, Faculty of Vocational Studies, Universitas Airlangga, Jl. Dharmawangsa Dalam Selatan No. 28-30, Kampus B Airlangga, Surabaya, 60115, East Java, Indonesia; 12 Department of Microbiology and Parasitology, Faculty of Veterinary Medicine, Universitas Pendidikan Mandalika, Jl. Pemuda No. 59A, Dasan Agung Baru, Mataram, 83125, West Nusa Tenggara, Indonesia

**Keywords:** antibiotic resistance, bats, *Escherichia coli*, extended-spectrum β-lactamase, guano, One Health, surveillance, zoonosis

## Abstract

The emergence and global dissemination of extended-spectrum β-lactamase (ESBL)-producing *Escherichia coli* represent a major public health concern. While antibiotic resistance in clinical and agricultural settings is well documented, the contribution of wildlife, particularly bats, to the spread of antimicrobial resistance (AMR) remains underexplored. Bats possess unique ecological traits – such as long-distance flight, longevity, and adaptability – that facilitate their role as potential reservoirs and vectors of antibiotic-resistant bacteria. This review synthesizes global findings on the occurrence, genetic characteristics, and transmission dynamics of ESBL-producing *E. coli* isolated from bats. Through a comprehensive literature review of studies conducted across five continents, we highlight the prevalence of multidrug-resistant *E. coli* in bat populations, with resistance profiles frequently including β-lactams (*bla*), aminoglycosides, tetracyclines, and fluoroquinolones. Notably, key ESBL genes such as *bla*CTX-M, *bla*TEM, *bla*SHV, and *bla*OXA have been identified in isolates from bat feces (guano), raising significant concern due to potential environmental contamination and zoonotic spillover. Risk factors such as habitat encroachment, anthropogenic waste exposure, and the agricultural use of bat guano further exacerbate the risk of ESBL transmission. Moreover, genomic comparisons suggest phylogenetic overlap between ESBL-producing *E. coli* from bats and those found in humans and livestock. Given these findings, bats warrant greater inclusion in One Health surveillance frameworks to trace AMR gene flow and develop targeted interventions. This review underscores the need for integrated AMR monitoring in wildlife, enhanced waste management policies, and stricter biosecurity to mitigate the public health risks associated with wildlife-origin ESBL dissemination.

## INTRODUCTION

The irrational use of antibiotics in humans [1, 2], companion animals [3, 4], livestock [5, 6], and aquaculture [7, 8] has significantly contributed to the emergence and proliferation of antibiotic-resistant bacteria (ARB), representing a major public health concern with substantial economic implications [9, 10]. From a One Health perspective, public health and environmental health are intrinsically linked, with wildlife playing an integral role in this interface [11]. Although wildlife are not typically subjected to direct antibiotic treatment, they can acquire resistant bacteria through environmental exposure – such as ingestion of contaminated food or water, migration from natural habitats to anthropogenically altered environments, or direct contact with domestic animals and humans [12]. Anthropogenic activities have greatly contributed to environmental degradation, including the destruction and fragmentation of wildlife habitats [13]. Such activities, including deforestation, hunting, and land conversion for agriculture and urban development, have disrupted bat habitats and brought them into closer proximity with human populations [14–17]. These human-induced disturbances facilitate the transmission of ARB within wildlife populations and pose significant threats under the One Health paradigm [18].

Bats may serve as valuable bioindicators for assessing regional patterns in antimicrobial resistance (AMR) prevalence and distribution [19]. Due to their exceptional flight capabilities, long lifespan, and ecological adaptability, bats are key species for understanding the ecological dynamics of AMR transmission. Consequently, bats are important research subjects for evaluating regional antibiotic resistance levels and mitigating its environmental dissemination [20]. Wildlife can function both as reservoirs and vectors of resistant bacteria, facilitating the spread of resistance across habitats and even continents through long-distance movement [21]. Gastrointestinal microbiota, such as bacilli and lactic acid bacteria, in bats exhibit antioxidant and pro-mutagenic gene expression profiles that enable a unique antiviral immune response. This adaptive immunity allows bats to mount a slow yet efficient response to infection, enabling them to tolerate or eliminate pathogens [22]. Gerbáčová *et al*. [23], Obodoechi *et al*. [24], and Gaeta *et al*. [25] have confirmed that bats harbor both Gram-negative and Gram-positive bacteria that display resistance to multiple antibiotic classes.

The first investigation into antibiotic resistance in bat-associated bacteria was conducted by Graves *et al*. [26], who collected samples from bats in West Java and Krakatau Island, Indonesia. The study revealed that Gram-negative bacteria, notably *Escherichia coli*, *Klebsiella*, and *Enterobacter*, were the predominant isolates and exhibited resistance to antibiotics such as ampicillin, trimethoprim, sulphamethoxazole, and cephalothin. The detection of antibiotic resistance in these bats is attributed to contamination from human fecal matter. Members of the *Enterobacteriaceae* family are among the most resistant and are capable of producing extended-spectrum β-lactamases (ESBLs) – enzymes first identified in 1983 that hydrolyze third- and fourth-generation β-lactam (*bla*) antibiotics, including cefotaxime [27]. A substantial body of research has since focused on ESBL-producing bacteria in animals, with particular attention to *E. coli* and *Klebsiella pneumoniae*.

Despite increasing awareness of AMR as a critical One Health concern, the role of wildlife – particularly bats – in the dissemination of ESBL-producing *E. coli* remains poorly understood. Current literature has extensively documented ESBL-associated *E. coli* in human and livestock populations; however, surveillance data on wildlife reservoirs are fragmented and geographically limited. While some studies have reported the presence of resistant *E. coli* in bats, few have comprehensively evaluated their global distribution, genetic characteristics, transmission dynamics, or ecological factors contributing to their persistence and spread. Furthermore, most existing research lacks comparative genomic analysis between bat-derived *E. coli* isolates and those found in humans or domesticated animals, which is essential for elucidating zoonotic potential. The underrepresentation of wildlife in AMR monitoring frameworks impedes our ability to trace resistance pathways and formulate effective mitigation strategies. Consequently, there is an urgent need to consolidate and synthesize the available evidence on the contribution of bats to the emergence and dissemination of ESBL-producing *E. coli* across ecological and geographical scales.

This review aims to synthesize current knowledge on the occurrence, distribution, resistance profiles, and public health implications of ESBL-producing *E. coli* isolated from bats globally. Specifically, it seeks to (i) evaluate the prevalence of ESBL-producing *E. coli* across different bat species and regions, (ii) describe the genetic determinants of resistance, including prevalent ESBL genes, (iii) explore ecological and anthropogenic factors influencing transmission dynamics, and (iv) assess the potential risks posed to humans, animals, and environmental health. By integrating global data and identifying research gaps, this review contributes to the development of a more comprehensive One Health surveillance framework and highlights the importance of including wildlife in AMR monitoring and control efforts.

## ANTIBIOTIC RESISTANCE MECHANISMS

Microorganisms are considered resistant when they exhibit significantly reduced susceptibility compared to isolates that remain sensitive. Bacterial resistance can arise through spontaneous genetic mutations or through the horizontal acquisition of resistance genes from external genetic sources [28]. Acquired resistance typically results from the uptake of resistance genes, chromosomal DNA mutations, or a combination of both mechanisms [29]. ARB and antibiotic resistance genes may be transmitted through both vertical and horizontal gene transfer (HGT) processes, including conjugation, transduction, and transformation [30].

Vertical gene transfer refers to the hereditary transmission of genetic material from parent to progeny during bacterial replication [31]. In contrast, HGT involves the transfer of genetic material between different bacterial species and represents the predominant pathway for the dissemination of antibiotic resistance, particularly through plasmid-mediated conjugation. Plasmids harbor genes with a high likelihood of being mobilized through mobile genetic elements (MGEs) [32]. These plasmids frequently encode traits that confer survival advantages, such as resistance to antibiotics, heavy metals, and disinfectants, as well as genes related to metabolism and virulence.

Resistance genes support bacterial survival through three principal mechanisms: (i) The production of ESBLs; (ii) alteration of intracellular antibiotic concentrations through efflux systems such as the tetracyclines A (*tetA*) pump; and (iii) protection of antibiotic targets, including genes such as *sul1* or *qnr* [33].

## HGT MECHANISM

Plasmid conjugation in bacteria functions as a mechanism for HGT, whereby MGEs facilitate the transfer of genetic material from one bacterial cell to another. This process is mediated by the type IV secretion system, which enables the mobilization of DNA from donor to recipient cells. Notably, conjugative plasmids can transfer genetic material without the requirement for specific receptors on recipient cells [34]. These plasmids often confer adaptive advantages to their bacterial hosts, including tolerance to high concentrations of disinfectants and heavy metals, as well as novel metabolic capabilities [35]. HGT facilitates rapid genetic diversification, enabling bacterial populations to adapt to selective pressures such as antibiotic exposure. Moreover, HGT contributes to infectious disease outbreaks by promoting biofilm formation and the dissemination of virulence factors [36].

Transduction refers to the phage-mediated transfer of genetic material from one bacterium to another. Bacteriophages are ubiquitous in the environment and are characterized by their resilience under a range of conditions, including alkaline and acidic environments, elevated temperatures, and exposure to chlorination or ultraviolet irradiation. These durable properties allow phages to persist in diverse ecosystems, where they form complex mutualistic relationships with bacteria over extended evolutionary timescales [37]. Experimental study by Tao *et al*. [38] in murine models has demonstrated that transduction can generate genetically diverse *E. coli* strains and contribute to the emergence of AMR within the gut microbiota.

Transformation occurs when bacteria uptake extracellular DNA from their environment, typically originating from lysed cells. This process involves a temporary alteration in cell membrane permeability to facilitate the incorporation of foreign DNA, thereby enabling the acquisition of novel genetic traits [39]. Unlike conjugation and transduction, transformation does not require viable donor cells; rather, it relies on environmental DNA from dead organisms [40].

Resistance genes are categorized based on the class of antibiotics they counteract, including *bla*, aminoglycosides (*aac*), and *tet*, among others [41]. In Enterobacteriaceae, one of the most clinically significant mechanisms of resistance involves the enzymatic hydrolysis of *bla* antibiotics – such as penicillins, monobactams, and cephalosporins – by ESBLs [42]. The first report of an ESBL-producing bacterial strain was documented in 1983 [43]. The ESBL enzyme family includes several variants such as Temoneira (TEM), sulfhydryl variable (SHV), cefotaxime-hydrolyzing β-lactamase (CTX-M), inhibitor-resistant TEM, complex mutant TEM-1, originally identified in *Klebsiella oxytoca* (OXA), guiana extended-spectrum (GES), commonly found in *Pseudomonas aeruginosa*, pseudomonas extended resistant (PER), Belgium extended β-lactamase, vietnam extended-spectrum β-lactamase (VEB), Tlahuica, and *Serratia fonticola* [44]. According to the classification by Bush-Jacoby-Medeiros, ESBLs are grouped functionally into three major categories, whereas the Ambler molecular classification divides ESBLs into four structural classes: Class A (e.g., TEM, SHV, and CTX-M), Class B (e.g., ESBLM-C, ESBLM-D, and OXA), Class C (e.g., ESBLCABRA), and Class D (e.g., PER, VEB, GES, and IBC) [45].

## EPIDEMIOLOGY OF *E. coli* RESISTANCE IN BATS

*E. coli* is widely used as an indicator organism for monitoring antibiotic resistance due to its inherent ability to readily transfer resistance genes to other bacterial strains [46]. It is a common commensal bacterium inhabiting the gastrointestinal tracts of both animals and humans [47]. However, certain strains of *E. coli* possess virulence factors capable of causing a range of clinical conditions, including gastroenteritis, pneumonia, septicemia, and cystitis [48]. The primary reservoir for the dissemination of *E. coli* is fecal matter. In bats, feces – commonly referred to as guano – serve as an environmental source of bacterial transmission. Bat guano is frequently employed in agriculture as an organic fertilizer due to its rich nutrient profile, which enhances soil fertility [49]. Nonetheless, its application as a biofertilizer raises public health concerns, as it may serve as a vector for foodborne pathogens and contribute to the contamination of livestock, water sources, and agricultural products [50].

Beyond its use in agriculture, guano from bats residing in urban parks, residential trees, and farmlands may facilitate bacterial dissemination as these animals forage at night or roost during the day in proximity to human activity [51]. The improper disposal and treatment of waste containing antibiotic residues, often termed “antibiotic pollution,” exacerbates the environmental burden of resistance [52]. There is a strong correlation between environmental antibiotic contamination and the emergence and persistence of resistant bacterial strains in polluted ecosystems [53]. The presence of antimicrobial-resistant bacteria in hosts – whether human, animal, or wildlife – can significantly reduce the effectiveness of available antibiotics and limit therapeutic options for bacterial infections in multiple species, including pets and livestock [54].

Aquatic and terrestrial ecosystems, particularly water and soil, play a critical role in the propagation of antibiotic resistance among wildlife [55]. Soil-dwelling invertebrates and microbial communities may harbor antibiotic residues or resistant bacteria, which can come into direct contact with or be ingested by foraging wildlife [56]. Human-driven activities contribute substantially to the interspecies transmission of AMR. Alarmingly, a considerable portion of the public remains unaware of their susceptibility to exposure or infection with resistant bacteria [57], let alone the potential for resistance transmission via wildlife reservoirs [58, 59]. Data on AMR in wildlife offer valuable insights for epidemiological assessments and are essential for informing future surveillance strategies aimed at understanding and mitigating resistance dissemination among wildlife populations [60].

Table 1 [23, 61–77] presents global data on antibiotic resistance profiles in *E. coli* isolated from bats, highlighting the diversity of resistance across different geographic regions.

**Table 1:** Epidemiology of antibiotic resistance of *Escherichia coli* in bats.

Continent	Country	Antibiotic resistance profile	Reference
Europe	Portugal	Ampicillin and streptomycin	[61]
	Portugal	Cefotaxime, *tet*, and ampicillin	[62]
	Poland	Kanamycin, sulfamethoxazole/trimethoprim, and streptomycin	[63]
	Portugal	Ampicillin, piperacillin, tazobactam, cephalexin, cefuroxime, cefixime, cefotaxime, cefepime, nalidic acid, ofloxacin, trimethoprim, trimethoprim/sulfamethoxazole	[64]
	Slovenia	Ciprofloxacin and Nalidix	[65]
America	Peru	Aztreonam, cefotaxime, cephalexin, and cefepime	[66]
	Brazil	Ampicillin, amoxicillin, and *tet*	[67]
Asia	Indonesia	Azitromycin, amoxicillin, *tet*, sulfamethoxazole/trimethroprim, ciprofloxacine, and gentamicin	[68]
	Indonesia	Ceftazidime	[69]
	Bangladesh	Cefepime and ampicillin	[70]
	Bangladesh	Amoxicillin and erythromycin	[71]
Africa	Nigeria	Penicillin, *tet*, sulfamethoxazole/trimethoprim, and gentamicin	[23]
	Nigeria	Amoxicillin, *tet*, augmentin, ceftriaxone, nitrofurantoin, gentamicin, cortimoxazole, ofloxacin, and pefloxacin	[72]
	Nigeria	Augmentin, cefuroxime, ceftazidime, amoxicillin, and cefotaxime	[73]
	Gabon	Amoxicillin, ampicillin, amoxicillin/clavulanic acid, ticarcillin, ticarcillin/clavulanic acid, piperacillin, cephalexin, cefoxitin, cefotaxime, cefpodoxime, ceftazidime, cefepime, aztreonam, ertapenem, amikacin, gentamycin, kanamycin, streptomycin, tobramycin, erythromycin, fosfomycin, *tet*, colistin, trimethropoietin/sulfamethoxazole, nalidixic acid, ciprofloxacin, and levofloxacin	[74]
	Nigeria	Augmentin, cefuroxime, ceftazidime, amoxicillin, and cefotaxime	[75]
Australia	Australia	Amoxicillin, *tet*, trimethoprim/sulfamethoxazole, *aac*, first- and third-generation cephalosporins	[76]
	Australia (South Australia)	Amoxicillin, amoxicillin-clavulanic acid, cephalosporin	[77]

*tet*=Tetracycline, *aac*=Aminoglycosides

### Europe

A total of 42 *E. coli* isolates obtained from bats in Portugal exhibited resistance to ampicillin and streptomycin, with resistance rates of 57.14% and 52.38%, respectively. In addition, the virulence gene fimA was detected in 21 of these isolates [61]. Another study by Garcês *et al*. [62] in Portugal reported that 9.6% of ESBL-producing *E. coli* isolated from *Tadarida teniotis* bats demonstrated resistance to cefotaxime, *tet*, and ampicillin [62]. In Poland, *E. coli* strains isolated from bats captured in the Lublin Upland exhibited the highest resistance to kanamycin (84.2%), followed by sulfamethoxazole/trimethoprim and streptomycin [63]. Furthermore, isolates from bats sampled in a Portuguese natural park showed resistance to a broad spectrum of antibiotics, including ampicillin, piperacillin, tazobactam, cephalexin, cefuroxime, cefixime, cefotaxime, cefepime, nalidixic acid, ofloxacin, trimethoprim, and trimethoprim/sulfamethoxazole [64].

*E. coli* is known to produce approximately 80 distinct lipoproteins involved in virulence, peptidoglycan synthesis and remodeling, cellular stress responses, and repair mechanisms. These lipoproteins contribute to the bacterium’s capacity to regulate antibiotic entry and maintain cellular stability [78]. Despite regulatory measures, the use of antibiotics such as ampicillin and *tet* remains prevalent in European animal production systems, particularly in swine farms, both for therapeutic and prophylactic purposes – practices that contravene current European regulations on antibiotic stewardship [79]. Ampicillin, a *bla* antibiotic, is inactivated by β-lactamases through hydrolysis of its β-lactam ring. Resistance to ampicillin is commonly associated with ESBL genes such as *bla*TEM, *bla*SHV, *bla*CTX-M, *bla*CMY, and *bla*OXA [80].

Interestingly, a study of 211 fruit-eating cave bats in Slovenia identified 185 *E. coli* isolates, all of which remained sensitive to ampicillin, *tet*, and chloramphenicol, but demonstrated resistance to ciprofloxacin and nalidixic acid [65].

### America

A longitudinal study conducted in Peru between 2015 and 2018 on *Desmodus rotundus* bats revealed a high prevalence of *E. coli* isolates resistant to multiple antibiotics, surpassing resistance levels observed in livestock from the same regions. All tested antibiotics – including third-generation cephalosporins such as aztreonam, cefotaxime, cephalexin, and cefepime– showed reduced efficacy [66]. Notably, the *E. coli* core genome multilocus sequence typing strain ST167 identified in 2018 matched strains collected from the same bat species 2 years prior. In addition, ST648 isolates identified from 2015 to 2017 displayed highly similar plasmid profiles across individuals sampled up to 100 km apart, suggesting long-term circulation and dissemination of colonizing clones among geographically separated bat populations [66].

In contrast, research conducted in Brazil yielded comparatively low resistance levels among *E. coli*, *K. oxytoca*, and *Staphylococcus* spp. isolates derived from bats residing in a national park. This was attributed to minimal anthropogenic influence in the protected area. Researchers recommended restricting tourist access to minimize human-induced environmental contamination [54]. However, Brazilian study by Sens-Junior *et al*. [67] near urban areas found that *E. coli* isolates from *Artibeus lituratus* bats exhibited resistance to ampicillin (36.36%), amoxicillin (36.36%), and *tet* (27.27%) [67].

Wildlife is increasingly exposed to antimicrobial compounds and resistant bacteria through anthropogenic sources such as sewage discharge, improper waste disposal, contaminated water bodies, predation on infected organisms, and proximity to intensive livestock operations [81]. Animal-derived food products are considered significant contributors to the dissemination of AMR among human populations and the environment [82]. In the Americas, approximately 65% of all antibiotics are sold for use in food-producing animals [83]. Resistant bacteria from this sector can spread through multiple pathways, including contaminated food products [84], occupational exposure [85], and environmental contamination [86], ultimately influencing the microbial ecology of wildlife habitats and increasing the risk of resistance transmission to and from wild animal populations.

### Asia

In Indonesia, initial studies on the antibiotic resistance of *E. coli* date back to 1988; however, research specifically targeting *E. coli* isolated from bats was only conducted in 2024. Bat specimens were collected from caves located near human settlements. Out of 135 collected samples, 97 *E. coli* isolates displayed resistance to multiple antibiotics, including azithromycin (38.1%), amoxicillin (24.7%), *tet* (24.7%), sulfamethoxazole/trimethoprim (22.6%), ciprofloxacin (14.4%), and gentamicin (1%) [68]. Another study conducted by Mustika *et al*. [69] found that 37% of *E. coli*-positive samples from 150 bat guano specimens exhibited multidrug-resistance (MDR), particularly to third-generation cephalosporins such as ceftazidime.

The use of antibiotics in Indonesian poultry farms to enhance animal performance is common practice [87]. These antibiotics are frequently employed as growth promoters, contributing to environmental accumulation and increasing the risk of resistance emergence [88].

In Bangladesh, 104 *E. coli* isolates were recovered from 369 bat guano samples. Among these, 28.18% were resistant to cefepime (16%) and ampicillin (13%) [70]. A separate investigation on *Rousettus leschenaulti* fruit bats revealed that all *E. coli* isolates were fully resistant to amoxicillin and erythromycin [71]. Similar to Indonesia, the majority of Bangladeshi poultry farmers lack adequate knowledge of appropriate livestock management and rational antibiotic use [89]. Wastewater discharge from poultry farms represents a significant source of AMR dissemination in the environment, particularly due to insufficient waste management and limited sanitation infrastructure in both rural and urban regions [90, 91].

### Africa

In Nigeria, all *E. coli* isolates obtained from bat fecal samples exhibited statistically significant resistance (p < 0.05) to amoxicillin, *tet*, and augmentin, with additional resistance observed against ceftriaxone, nitrofurantoin, gentamicin, cotrimoxazole, ofloxacin, and pefloxacin. Notably, 90% of the isolates demonstrated MDR [72]. Resistance patterns in *E. coli* strains from bats and broiler chickens were found to be similar, likely due to inadequate sanitation practices on intensive poultry farms [92]. In 2021, Nigeria reported increased resistance in several antibiotic classes: penicillin (48.6%), *tet* (37.1%), sulfamethoxazole/trimethoprim (22.9%), and gentamicin (20.0%) [23]. A follow-up study by Aladejana *et al*. [73] in 2022 further confirmed a notable rise in *E. coli* resistance to augmentin, cefuroxime, ceftazidime, amoxicillin, and cefotaxime.

The spatial proximity between bats and humans or livestock significantly correlates with resistance profiles, particularly in regions where similar antibiotics are used in both veterinary and human medicine [93]. Supporting this, a 2017 study in the Republic of Congo reported no resistance among 39 *E. coli* isolates from 50 bat guano samples. The absence of resistance was attributed to abundant food resources in the bats’ natural habitat, which minimized their contact with human settlements and potential antibiotic exposure [94].

In Gabon, isolates of *E. coli*, *K. pneumoniae*, and *Enterobacter cloacae* from *Epomops franqueti* and *Megaloglossus woermanni* bats residing in the Makokou forest were resistant to a wide range of antibiotics, including amoxicillin, ampicillin, amoxicillin/clavulanic acid, ticarcillin, ticarcillin/clavulanic acid, piperacillin, cephalexin, cefoxitin, cefotaxime, cefpodoxime, ceftazidime, cefepime, aztreonam, ertapenem, amikacin, gentamicin, kanamycin, streptomycin, tobramycin, erythromycin, fosfomycin, *tet*, colistin, trimethoprim/sulfamethoxazole, nalidixic acid, ciprofloxacin, and levofloxacin [74]. Similarly, *E. coli* isolates from the feces of *Eidolon helvum* in Osun State, Nigeria, showed resistance to augmentin, cefuroxime, ceftazidime, amoxicillin, and cefotaxime [75].

### Australia

In Australia, the prevalence of antibiotic-resistant *E. coli* in bats remains relatively low. Only 3.7% (12 out of 318) of the isolates exhibited resistance, though these were 100% resistant to amoxicillin, *tet*, trimethoprim/sulfamethoxazole, *aac*, and both first- and third-generation cephalosporins [76]. In addition, 53 *E. coli* isolates from *Pteropus poliocephalus* bats rescued in South Australia demonstrated resistance to amoxicillin (77.4%, n = 41), amoxicillin-clavulanic acid (24.5%, n = 13), and cephalosporins (11.3%, n = 6) [77]. The close proximity of agricultural and livestock zones in Australia increases the likelihood of AMR bacterial contamination, as evidenced by the overlapping resistance profiles observed in *E. coli* strains from both wildlife and domestic animals [95].

## RISK FACTORS FOR ESBL-PRODUCING *E. coli*

The incidence of antibiotic-resistant *E. coli* continues to rise annually, posing significant threats to both human and animal health. These resistant strains are increasingly detected in healthcare facilities and community settings [96]. *E. coli* is one of the primary pathogens implicated in urinary tract infections (UTIs), and approximately 70%–90% of clinical isolates in community settings have demonstrated resistance and the ability to produce ESBLs. The global dissemination of MDR ESBL-producing *E. coli* is of particular concern to the World Health Organization (WHO), given the difficulty in treatment and control [97].

Wildlife, including bats, functions as important reservoirs and vectors for these resistant bacteria due to their ecological overlap with human populations [98]. Treating infections caused by ESBL-producing bacteria becomes increasingly challenging due to the presence of resistance genes that render multiple antibiotic classes – such as trimethoprim, *aac*, cephalosporins, and macrolides – ineffective [99].

Bats exhibit biological traits that enhance their role in the environmental dissemination of ESBL-producing bacteria. Their exceptional longevity relative to body size [100], robust immune tolerance to pathogens [101], and high mobility through long-distance flight [102] contribute to their potential as widespread reservoirs. Bats are classified into two primary suborders: Megachiroptera (fruit-eating) and Microchiroptera (insectivorous) [103]. Urban expansion and habitat destruction have forced many bats to shift from forest ecosystems to urban and peri-urban environments. Insectivorous bats are commonly found near artificial lighting where insects congregate, while frugivorous bats often forage in cultivated or residential areas due to deforestation and declining wild fruit availability [104–106].

Human exposure to ESBL-producing *E. coli* from bats may occur through ingestion of contaminated fruits or water, direct contact with unprocessed guano, or recreational activities involving contaminated natural water sources [107, 108]. Populations residing in or near AMR hotspots are at greater risk of colonization or infection by ESBL-producing strains compared to individuals living in less impacted regions [109]. Phylogenetic analyses have revealed genetic similarities in ESBL determinants between wildlife and human-associated *E. coli*, suggesting potential cross-species transmission [110].

Furthermore, identical resistance genes have been detected across geographically distant populations, implying long-range dissemination potentially driven by bat migration or human-mediated factors such as global travel. For instance, a study by Hayer *et al*. [111] in Chile documented overlapping AMR gene profiles, virulence genes, and plasmid replicons in livestock, wildlife, and domestic dogs within a 15 km radius. Bats residing in seemingly pristine forest environments can also harbor resistant bacteria due to complex transmission pathways, including environmental contamination from tourism and inadequate waste disposal practices [112]. Table 2 [23, 62, 63, 66, 69, 73, 76, 113, 114] shows the ESBL *E. coli* data from bat isolates from various regions.

**Table 2:** ESBL-producing *Escherichia coli* from bats.

Continent	Country	ESBL resistance gene	Reference
Europe	Portugal	*bla*CTX-M-1, *bla*CTX-M-3, *bla*SHV, *bla*TEM, *bla*OXA, *bla*CTX-M-9, *bla*CTX-M	[62]
	Poland	*bla*CTX-M-3, *bla*CTX-M-5, *bla*TEM-1	[63]
	Portugal	*bla*CTX-M-1, *bla*CTX-M-32, *bla*CTX-M-14, *bla*SHV-12	[113]
America	Peru	*bla*CTX-M-55, *bla*CTX-M-15, *bla*CTX-M-65, *bla*CTX-M-3, and *bla*CTX-M-14	[66]
Asia	Indonesia	*bla*TEM	[69]
		*bla*TEM	[114]
Africa	Southeast Nigeria	*bla*CTX-M-15, *bla*TEM	[23]
	Nigeria	*bla*TEM	[73]
Australia	Australia	*bla*NDM, *bla*CTX-M-27	[76]

ESBL=Extended-spectrum β-lactamase, *bla*=β-lactams, TEM=Temoneira, SHA=Sulfhydryl variable, CTX-M=Cefotaxime-hydrolyzing β-lactamase, OXA=Originally identified in *Klebsiella oxytoca*, NDM=New Delhi Metallo-β-lactamase

Several ESBL genes found in bats – such as *bla*CTX-M, *bla*SHV, *bla*TEM, and *bla*OXA – are also frequently detected in humans and domestic animals. In Portugal, for example, *bla*CTX-M-1, *bla*SHV, *bla*TEM, and *bla*OXA have been identified in sheep [115], pigs [116], poultry [117], meat products [118], and human clinical isolates [119], as reported by Sabença *et al*. [64] and Garcês *et al*. [62]. Similar trends have been observed in Peru [120–122], where *bla*TEM is prevalent among both livestock and human populations [123–126]. In Nigeria, *bla*CTX-M and *bla*TEM have been detected across wildlife, livestock, and human samples, highlighting the widespread distribution of these resistance determinants [127–130]. In addition, in Australia, *E. coli* strains from bats have been shown to harbor various plasmid-mediated resistance genes, including *aacA34, aadA1, aadA2*, *bla*OXA-2, *bla*OXA-21, *dfrA1, dfrA5, dfrA21, qacF*, and *qacH* [93].

## PUBLIC HEALTH IMPACT

*E. coli* is a commensal bacterium naturally present in the gastrointestinal tracts of both humans and animals [131]. ESBL genes found in bats exhibit considerable similarity to those commonly identified in *E. coli* strains isolated from humans and domestic animals. ESBL-producing *E. coli* has been documented across various ecological contexts, including human populations, livestock, wildlife, and along the food supply chain [132]. Resistance levels are reportedly higher in female and juvenile animals, a trend likely attributable to the prophylactic use of antibiotics during gestation, therapeutic applications during lactation, and routine administration as growth promoters in young animals [133].

The presence of ESBL-producing *E. coli* in humans significantly complicates the treatment of infections such as UTIs, pneumonia, bacteremia, and tuberculosis. In addition, the efficacy of medical interventions such as cancer chemotherapy, organ transplantation, intubation, and catheterization is compromised due to limited treatment options [134–138]. Consequently, mortality associated with ESBL-related infections continues to rise worldwide, with carbapenems often becoming the last line of defense in such cases.

Global prevalence data indicate that ESBL carriage rates are highest in Southeast Asia (46%, 95% CI: 29%–63%), followed by Southern Europe (6%, 95% CI: 1%–12%), Northern Europe (4%, 95% CI: 2%–6%), Central Europe (3%, 95% CI: 1%–5%), South America (3%, 95% CI: 0%–7%), and North America (2%, 95% CI: 1%–5%) [139]. Environmental contamination – particularly of aquatic systems – through urine and feces from humans and animals carrying ESBL-producing strains further facilitates the widespread dissemination of these organisms [140]. As *E. coli* serves as a key indicator of fecal contamination, its detection plays a central role in food and water safety surveillance [141].

The economic burden of ESBL-producing *E. coli* is substantial, particularly when food contamination occurs, resulting in large-scale financial losses across agricultural and public health sectors [67]. Surveillance of ESBL in wildlife is especially critical, as these populations often move unpredictably across regions, making containment difficult. Monitoring efforts in free-ranging species are essential not only for mapping the geographic distribution of resistance genes but also for identifying potential reservoirs and transmission pathways relevant to human health [142].

From a clinical standpoint, patients infected with ESBL-producing bacteria tend to require prolonged hospital stays and intensive care, thereby incurring higher medical costs [23]. Recognizing the severity of the issue, the WHO has categorized antibiotic resistance as a pressing global health threat [143]. Current estimates attribute over 700,000 deaths annually to antibiotic-resistant infections, with projections suggesting that this figure could escalate to 10 million deaths/year by 2050 if current trends persist [144].

Regionally, the prevalence of ESBL-producing *E. coli* in Asia has risen sharply. Between 2002 and 2011, resistance rates in China increased from 36.1% to 68.1%. In India, the prevalence reached 79%, followed by Vietnam (34.4%), Thailand (50.8%), Singapore and South Korea (33.3%), Hong Kong (17.8%), Taiwan (12.7%), and the Philippines (17.0%) [145].

## ESBL CONTROL

Enhanced biosecurity practices in animal husbandry have proven to be effective strategies for mitigating the transmission of ESBL-producing *E. coli* between livestock and farm workers [146]. Strict adherence to hygiene protocols and minimizing exposure to known or potential carriers are essential measures for reducing the spread of ESBL-producing organisms in both clinical and community environments. Regular disinfection of hospital environments, including incubators, medical devices, and frequently contacted surfaces, plays a critical role in curbing nosocomial transmission [147].

In addition to local interventions, effective containment of AMR – including ESBL – relies on robust global surveillance systems. Programs such as the Global Antimicrobial Resistance and Use Surveillance System (GLASS) and the World Organisation for Animal Health (formerly Office International des Epizooties) provide coordinated platforms for tracking resistance trends. These efforts are further supported by regional initiatives including the European Antimicrobial Resistance Surveillance Network (EARS-Net), the Latin American Antimicrobial Resistance Surveillance Network (ReLAVRA), and the Central Asian and Eastern European Surveillance of Antimicrobial Resistance, which collectively strengthen international monitoring and response capacity [148].

In agricultural contexts, the composting of bat guano before use as fertilizer is recommended as a practical method to reduce microbial loads and limit the environmental dissemination of ARB [149].

Furthermore, novel therapeutic strategies are being developed to manage antibiotic resistance. These include combination therapies involving *bla* antibiotics with other antibiotic classes [150], the co-administration of β-lactamase inhibitors, and the synergistic use of antibiotics with biocides. Innovative approaches such as the use of phytochemicals, small-molecule inhibitors, and RNA interference are also being investigated as alternative or adjunctive treatments to combat resistant pathogens [151, 152]. These emerging solutions offer promising avenues to counter the growing threat of MDR bacteria.

## CONCLUSION

The emergence of ESBL-producing *E. coli* as a critical public health concern underscores the need for an expanded One Health surveillance framework that includes wildlife reservoirs such as bats. This review synthesizes global evidence demonstrating that bats harbor ESBL-producing *E. coli* strains with genetic, phenotypic, and resistance profiles comparable to those found in humans, domestic animals, and environmental sources. The detection of key resistance genes – such as *bla*CTX-M, *bla*TEM, *bla*SHV, and *bla*OXA – in bat-associated isolates highlights the zoonotic potential and ecological interconnectedness of AMR transmission pathways.

Risk factors contributing to the spread of ESBL-producing *E. coli* from bats include habitat encroachment, environmental contamination from anthropogenic waste, agricultural misuse of antibiotics, and inadequate waste management in rural and peri-urban areas. The high mobility, ecological plasticity, and longevity of bats amplify their role as long-range vectors, capable of disseminating resistant bacteria across ecosystems and geographical barriers.

The public health implications are profound. ESBL-producing *E. coli* contributes to increased morbidity, mortality, and treatment costs, particularly in infections for which therapeutic options are severely limited. Surveillance data indicate rising ESBL prevalence across continents, with Asia and parts of Europe showing the highest burden. Moreover, the economic consequences extend beyond healthcare to food safety and trade, especially where contaminated guano is used as fertilizer.

Effective mitigation requires a multifaceted approach involving enhanced biosecurity in livestock systems, improved sanitation, wildlife monitoring, and global coordination through surveillance networks such as GLASS, EARS-Net, and ReLAVRA. Innovative treatment modalities, including enzyme inhibitors, phytochemicals, and RNA interference, offer promising avenues for addressing therapeutic challenges.

Bats represent a significant but often overlooked reservoir of ESBL-producing *E. coli*. Incorporating wildlife into AMR surveillance and response strategies is essential to curbing the global spread of resistance genes. Future research should focus on genomic comparisons, transmission modeling, and ecological risk assessments to inform evidence-based interventions at the human–animal–environment interface.

## AUTHORS’ CONTRIBUTIONS

ALDA, ARK, RZA, DAA, and IBM: Drafted the manuscript. SMY, RZA, FNAEPD, WW, and AW: Revised and edited the manuscript. MHE, ALDA, HP, WW, BB, and WT: Drafted and critically revised the manuscript. RZA, ALDA, KHPR, and WW: Edited the references. All authors have read and approved the final manuscript.

## References

[1] Nazwar T.A, Saputra B.R, Retnoningsih D, Bal'afif F, Wardhana D.W (2023). Prevalence and antibiotic resistance of bacteria isolated from cerebrospinal fluid of neurosurgical patients in Malang, Indonesia. Mod. Med.

[2] Sinto R, Lie K.C, Setiati S, Suwarto S, Nelwan E.J, Karyanti M.R, Karuniawati A, Djumaryo D.H, Prayitno A, Sumariyono S, Sharland M, Moore C.E, Hamers R.L, Day N.P.J, Limmathurotsakul D (2024). Diagnostic and antibiotic use practices among COVID-19 and non-COVID-19 patients in the Indonesian National Referral Hospital. PLoS One.

[3] Farizqi M.T.I, Effendi M.H, Adikara R.T.S, Yudaniayanti I.S, Putra G.D.S, Khairullah A.R, Kurniawan S.C, Silaen O.S.M, Ramadhani S, Millannia S.K, Kaben S.E, Waruwu Y.K.K (2023). Detection of extended-spectrum -lactamase-producing *Escherichia coli* genes isolated from cat rectal swabs at Surabaya Veterinary Hospital, Indonesia. Vet. World.

[4] Khairullah A.R, Sudjarwo S.A, Effendi M.H, Ramandinianto S.C, Gelolodo M.A, Widodo A, Riwu K.H.P, Kurniawati D.A (2023). Pet animals as reservoirs for spreading methicillin-resistant *Staphylococcus aureus* to human health. J. Adv. Vet. Anim. Res.

[5] Dameanti F.N.A.E.P, Yanestria S.M, Effendi M.H, Plumeriastuti H, Tyasningsih W, Ugbo E.N, Sutrisno R, Safri M.A.A.S (2023). Identification of *bla*SHV and *bla*TEM extended spectrum beta-lactamase genes in *Klebsiella pneumoniae* in the dairy wastewater, East Java Province, Indonesia. Biodiversitas.

[6] Prayudi S.K.A, Effendi M.H, Lukiswanto B.S, Az Zahra R.L, Benjamin M.I, Kurniawan S.C, Khairullah A.R, Silaen O.S.M, Lisnanti E.F, Baihaqi Z.A, Widodo A, Riwu K.H.P (2023). Detection of genes on *Escherichia coli* producing extended spectrum -lactamase isolated from the small intestine of ducks in traditional markets Surabaya City, Indonesia. J. Adv. Vet. Res.

[7] Naudet J, d'Orbcastel E.R, Bouvier T, Godreuil S, Dyall S, Bouvy S, Rieuvilleneuve F, Restrepo-Ortiz C.X, Bettarel Y, Auguet J.C (2023). Identifying macroplastic pathobiomes and antibiotic resistance in a subtropical fish farm. Mar. Pollut. Bull.

[8] Gao Z, Piao Y, Hu B, Yang C, Zhang X, Zheng Q, Cao J (2023). Investigation of antibiotic resistance genotypic and phenotypic characteristics of marine aquaculture fish carried in the Dalian area of China. Front. Microbiol.

[9] Riwu K.H.P, Rantam F.A, Effendi M.H, Khairullah A.R, Widodo A, Kurniawan S.C, Ugbo E.N (2022). Molecular detection of extended spectrum -lactamase producing *Klebsiella pneumoniae* from wild deer. Biodiversitas.

[10] Hossain M.J, Jabin N, Ahmmed F, Sultana A, Abdur Rahman S.M, Islam M.R (2023). Irrational use of antibiotics and factors associated with antibiotic resistance:Findings from a cross-sectional study in Bangladesh. Health Sci. Rep.

[11] Łopucki R, Stępień-Pyśniak D, Christensen H, Kubiński K, Lenarczyk E, Martinez-de-Tejada G, Kitowski I, Masłyk M (2024). Interspecies transmission of antimicrobial-resistant bacteria between wild birds and mammals in urban environment. Vet. Microbiol.

[12] Ramos C.A, Ferreira J.C, Ballaben A.S, Filho R.A.C.P, Darini A.L.D.C (2024). Analysis of antibiotic resistance in gram-negative bacilli in wild and exotic healthy birds in Brazil:A warning sign. Vet. Microbiol.

[13] Meramo K, Ovaskainen O, Bernard E, Silva C.R, Laine V.N, Lilley T.M (2022). Contrasting effects of chronic anthropogenic disturbance on activity and species richness of insectivorous bats in neotropical dry forest. Front. Ecol. Evol.

[14] Nahar N, Asaduzzaman M, Mandal U.K, Rimi N.A, Gurley E.S, Rahman M, Garcia F, Zimicki S, Sultana R, Luby S.P (2020). Hunting bats for human consumption in Bangladesh. Ecohealth.

[15] Pretorius M, Markotter W, Keith M (2021). Assessing the extent of land-use change around important bat-inhabited caves. BMC Zool.

[16] Rulli M.C, D'Odorico P, Galli N, Hayman D.T.S (2021). Land-use change and the livestock revolution increase the risk of zoonotic coronavirus transmission from rhinolophid bats. Nat. Food.

[17] Jackson R.T, Webala P.W, Ogola J.G, Lunn T.J, Forbes K.M (2023). Roost selection by synanthropic bats in rural Kenya:Implications for human-wildlife conflict and zoonotic pathogen spillover. R. Soc. Open Sci.

[18] Hu R, Ren M, Liang S, Zou S, Li D (2024). Effects of antibiotic resistance genes on health risks of rivers in habitat of wild animals under human disturbance-based on analysis of antibiotic resistance genes and virulence factors in microbes of river sediments. Ecol. Evol.

[19] De Silva O, Liyanagunawardena N, Pabasara A.B.S, Daluwatta D.L, Wickramasinghe K.W.D.M.Y.W, Weerasooriya K.M.S.G, Wijemuni M.I, Dissanayake D.M.S.N.B, De Alwis P.S, Samarakoon N, Priyantha M.A.R (2023). Antimicrobial resistance including extended spectrum beta lactamases (ESBL) and carbapenemase in feacal *E. coli* isolates from bats. Sri Lanka Vet. J.

[20] Stapelfeldt B, Tress C, Koch R, Tress J, Kerth G, Scheuerlein A (2023). Long-term field study reveals that warmer summers lead to larger and longer-lived females only in northern populations of Natterer's bats. Oecologia.

[21] Plaza-Rodríguez C, Alt K, Grobbel M, Hammerl J.A, Irrgang A, Szabo I, Stingl K, Schuh E, Wiehle L, Pfefferkorn B, Naumann S, Kaesbohrer A, Tenhagen B.A (2021). Wildlife as sentinels of antimicrobial resistance in Germany?. Front. Vet. Sci.

[22] Popov I.V, Mazanko M.S, Kulaeva E.D, Golovin S.N, Malinovkin A.V, Aleshukina I.S, Aleshukina A.V, Prazdnova E.V, Tverdokhlebova T.I, Chikindas M.L, Ermakov A.M (2021). Gut microbiota of bats:Pro-mutagenic properties and possible frontiers in preventing emerging disease. Sci. Rep.

[23] Gerbáčová K, Maliničová L, Kisková J, Maslišová V, Uhrin M, Pristaš P (2020). The faecal microbiome of building-dwelling insectivorous bats (*Myotis myotis* and *Rhinolophus hipposideros*) also contains antibiotic-resistant bacterial representatives. Curr. Microbiol.

[24] Obodoechi L.O, Carvalho I, Chenouf N.S, Martínez-Álvarez S, Sadi M, Nwanta J.A, Chah K.F, Torres C (2021). Antimicrobial resistance in *Escherichia coli* isolates from frugivorous (*Eidolon helvum*) and insectivorous (*Nycteris hispida*) bats in Southeast Nigeria, with detection of CTX-M-15 producing isolates. Comp. Immunol. Microbiol. Infect. Dis.

[25] Gaeta N.C, Brito J.E.C, Batista J.M.N, de Mello B.G.V, Dias R.A, Heinemann M.B (2023). Bats are carriers of antimicrobial-resistant Staphylococcaceae in their skin. Antibiotics (*Basel*).

[26] Graves S, Kennelly-Merrit S, Tidemann C, Rawlinson P, Harvey K, Thornton I (1988). Antibiotic-resistance patterns of enteric bacteria of wild mammals on the Krakatau Islands and West Java, Indonesia. Philos. Trans. R. Soc. Lond. B Biol. Sci.

[27] Homeier-Bachmann T, Schütz A.K, Dreyer S, Glanz J, Schaufler K, Conraths F.J (2022). Genomic analysis of ESBL-producing *E. coli* in wildlife from North-Eastern Germany. Antibiotics (*Basel*).

[28] Dugassa J, Shukuri N (2017). Review on antibiotic resistance and its mechanism of development. J. Health Med. Nurs.

[29] Khameneh B, Diab R, Ghazvini K, Bazzaz B.S.F (2016). Breakthroughs in bacterial resistance mechanisms and the potential ways to combat them. Microb. Pathog.

[30] Zhang G, Li W, Chen S, Zhou W, Chen J (2020). Problems of conventional disinfection and new sterilization methods for antibiotic resistance control. Chemosphere.

[31] Li X, Mowlaboccus S, Jackson B, Cai C, Coombs G.W (2024). Antimicrobial resistance among clinically significant bacteria in wildlife:An overlooked one health concern. Int. J. Antimicrob. Agents.

[32] Vázquez L, Srednik M.E, Rodríguez J, Flórez A.B, Mayo B (2023). Antibiotic resistance/susceptibility profiles of *Staphylococcus equorum* strains from cheese, and genome analysis for antibiotic resistance genes. Int. J. Mol. Sci.

[33] Palomino A, Gewurz D, DeVine L, Zajmi U, Moralez J, Abu-Rumman F, Smith R.P, Lopatkin A.J (2023). Metabolic genes on conjugative plasmids are highly prevalent in *Escherichia coli* and can protect against antibiotic treatment. ISME J.

[34] Mayorga-Ramos A, Zúñiga-Miranda J, Carrera-Pacheco S.E, Barba-Ostria C, Guamán L.P (2023). CRISPR-Cas-based antimicrobials:Design, challenges, and bacterial mechanisms of resistance. ACS Infect. Dis.

[35] Bobate S, Mahalle S, Dafale N.A, Bajaj A (2023). Emergence of environmental antibiotic resistance:Mechanism, monitoring and management. Environ. Adv.

[36] Lerminiaux N.A, Cameron A.D (2019). Horizontal transfer of antibiotic resistance genes in clinical environments. Can. J. Microbiol.

[37] Michaelis C, Grohmann E (2023). Horizontal gene transfer of antibiotic resistance genes in biofilms. Antibiotics (*Basel*).

[38] Tao S, Chen H, Li N, Wang T, Liang W (2022). The spread of antibiotic resistance genes *in vivo* model. Can. J. Infect. Dis. Med. Microbiol.

[39] Carvalho G, Fouchet D, Danesh G, Godeux A.S, Laaberki M.H, Pontier D, Charpentier X, Venner S (2020). Bacterial transformation buffers environmental fluctuations through the reversible integration of mobile genetic elements. mBio.

[40] Virolle C, Goldlust K, Djermoun S, Bigot S, Lesterlin C (2020). Plasmid transfer by conjugation in gram-negative bacteria:From the cellular to the community level. Genes (*Basel*).

[41] Jian Z, Zeng L, Xu T, Sun S, Yan S, Yang L, Huang Y, Jia J, Dou T (2021). Antibiotic resistance genes in bacteria:Occurrence, spread, and control. J. Basic Microbiol.

[42] Urban-Chmiel R, Marek A, Stępień-Pyśniak D, Wieczorek K, Dec M, Nowaczek A, Osek J (2022). Antibiotic resistance in bacteria-A review. Antibiotics (Basel).

[43] Knothe H, Shah P, Krcmery V, Antal M, Mitsuhashi S (1983). Transferable resistance to cefotaxime, cefoxitin, cefamandole and cefuroxime in clinical isolates of *Klebsiella pneumoniae* and *Serratia marcescens*. Infection.

[44] Castanheira M, Simner P.J, Bradford P.A (2021). Extended-spectrum -lactamases:An update on their characteristics, epidemiology and detection. JAC Antimicrob. Resist.

[45] Husna A, Rahman M.M, Badruzzaman A.T.M, Sikder M.H, Islam M.R, Rahman M.T, Alam J, Ashour H.M (2023). Extended-spectrum -lactamases (ESBL):Challenges and opportunities. Biomedicines.

[46] Widodo A, Lamid M, Effendi M.H, Khailrullah A.R, Kurniawan S.C, Silaen O.S.M, Riwu K.H.P, Yustinasari L.R, Afnani D.A, Dameanti F.N.A.E.P, Ramandinianto S.C (2023). Antimicrobial resistance characteristics of multidrug resistance and extended-spectrum beta-lactamase producing *Escherichia coli* from several dairy farms in Probolinggo, Indonesia. Biodiversitas.

[47] Kristianingtyas L, Effendi M.H, Witaningrum A.M, Wardhana D.K, Ugbo E.N (2021). Prevalence of extended-spectrum -lactamase-producing *Escherichia coli* in companion dogs in animal clinics, Surabaya, Indonesia. Int. J. One Health.

[48] Effendi M.H, Faridah H.D, Wibisono F.M, Wibisono F.J, Nisa N, Fatimah and Ugbo E.N (2022). Detection of virulence factor encoding genes on *Escherichia coli* isolated from broiler chicken in Blitar District, Indonesia. Biodiversitas.

[49] Barolia S.K, Singh P (2020). Bat (*Pteropus giganteus*) plays an important role in many ways for society and farmers:A review. Glob. J. Food Sci. Nutr.

[50] Dimkić I, Fira D, Janakiev T, Kabić J, Stupar M, Nenadić M, Unković N, Grbić M.L (2021). The microbiome of bat guano:For what is this knowledge important?. Appl. Microbiol. Biotechnol.

[51] Asai T, Sugiyama M, Omatsu T, Yoshikawa M, Minamoto T (2022). Isolation of extended-spectrum -lactamase-producing *Escherichia coli* from Japanese red fox (*Vulpes vulpes japonica*). Microbiologyopen.

[52] Barathe P, Kaur K, Reddy S, Shriram V, Kumar V (2024). Antibiotic pollution and associated antimicrobial resistance in the environment. J. Hazard. Mater. Lett.

[53] Addis T.Z, Adu J.T, Kumarasamy M, Demlie M (2024). Occurrence of trace-level antibiotics in the Msunduzi river:An investigation into South African environmental pollution. Antibiotics (Basel).

[54] Cláudio V.C, Gonzalez I, Barbosa G, Rocha V, Moratelli R, Rassy F (2018). Bacteria richness and antibiotic-resistance in bats from a protected area in the Atlantic Forest of Southeastern Brazil. PLoS One.

[55] Lepper H.C, Woolhouse M.E.J, van Bunnik B.A.D (2022). The role of the environment in dynamics of antibiotic resistance in humans and animals:A modelling study. Antibiotics (*Basel*).

[56] Kuppusamy S, Venkateswarlu K, Megharaj M, Lee Y.B (2024). Risks of veterinary antibiotics contamination in Indian organic farmlands:A reality unfolded. Environ. Adv.

[57] McCullough A.R, Parekh S, Rathbone J, Del Mar C.B, Hoffmann T.C (2016). A systematic review of the public's knowledge and beliefs about antibiotic resistance. J. Antimicrob. Chemother.

[58] Hiltunen T, Virta M, Laine A.L (2017). Antibiotic resistance in the wild:An eco-evolutionary perspective. Philos. Trans. R. Soc. Lond. B Biol. Sci.

[59] Vittecoq M, Godreuil S, Prugnolle F, Durand P, Brazier L, Renaud N, Arnal A, Aberkane S, Jean-Pierre H, Gauthier-Clerc M, Thomas F, Renaud F (2016). Antimicrobial resistance in wildlife. J. Appl. Ecol.

[60] Ramey A.M, Ahlstrom C.A (2020). Antibiotic resistant bacteria in wildlife:Perspectives on trends, acquisition and dissemination, data gaps, and future directions. J. Wildl. Dis.

[61] Garcês A, Correia S, Silva V, Pereira J.E, Amorim F, Igrejas G, Poeta P (2019). Detection of antimicrobial resistance in faecal *Escherichia coli* from European free-tailed bats (*Tadarida teniotis*) in Portugal. Acta Chiropt.

[62] Garcês A, Correia S, Amorim F, Pereira J.E, Igrejas G, Poeta P (2019). First report on extended-spectrum beta-lactamase (ESBL) producing *Escherichia coli* from European free-tailed bats (*Tadarida teniotis*) in Portugal:A one-health approach of a hidden contamination problem. J. Hazard Mater.

[63] Nowakiewicz A, Zięba P, Gnat S, Trościańczyk A, Osińska M, Łagowski D, Kosior-Korzecka U, Puzio I (2020). Bats as a reservoir of resistant *Escherichia coli*:A methodical view. Can we fully estimate the scale of resistance in the reservoirs of free-living animals?. Res. Vet. Sci.

[64] Sabença C, Igrejas G, Poeta P, Robin F, Bonnet R, Beyrouthy R (2021). Multidrug resistance dissemination in *Escherichia coli* isolated from wild animals:Bacterial clones and plasmid complicity. Microbiol. Res.

[65] Skok S, Kogovšek B, Šturm S, Avguštin J.A, Mulec J (2020). Antimicrobial resistant *Escherichia coli* from karst waters, surfaces and bat guano in Slovenian caves. Acta Cardiol.

[66] Benavides J.A, Godreuil S, Opazo-Capurro A, Mahamat O.O, Falcon N, Oravcova K, Streicker D.G, Shiva C (2022). Long-term maintenance of multidrug-resistant *Escherichia coli* carried by vampire bats and shared with livestock in Peru. Sci. Total Environ.

[67] Sens-Junior H, Trindade W.A, Oliveira A.F, Zaniolo M.M, Serenini G.F, Araujo-Ceranto J.B, Gonçalves D.D, Germano R.M (2018). Bacterial resistance in bats from the Phyllostomidae family and its relationship with unique health. Pesq. Vet. Bras.

[68] Agustin A.L.D, Dameanti F.N.A.E.P, Effendi M.H, Tyasningsih W, Khairullah A.R, Kurniawan S.C, Moses I.B, Hasib A, Mustika Y.R, Kinasih K.N (2024). Multidrug resistance to antibiotics in *Escherichia coli* bacteria isolated from bats on Lombok Island, Indonesia. J. Adv. Vet. Res.

[69] Mustika Y.R, Kinasih K.N, Effendi M.H, Puspitasari Y, Kurniawan S.C, Khairullah A.R, Samodra M.E.E, Hasib A, Agustin A.L.D, Moses I.B, Silaen O.S.M (2024). Molecular detection of extended-spectrum -lactamase-producing *Escherichia coli* from bat caves on Lombok Island. Open Vet. J.

[70] Talukdar P.K, Rahman M, Rahman M, Nabi A, Islam Z, Hoque M.M, Endtz H.P, Islam M.A (2013). Antimicrobial resistance, virulence factors and genetic diversity of *Escherichia coli* isolates from household water supply in Dhaka, Bangladesh. PLoS One.

[71] Islam M.N (2014). Prevalence and Antibiogram of *E. coli* and *Salmonella* spp. Isolates in Small Fruits Bat (*Rousettus leschenaulti*) and Associated Public Health Risk in Bangladesh. Chittagong Veterinary and Animal Sciences University, Khulshi-Ctg.

[72] Oluduro A.O (2012). Antibiotic-resistant commensal *Escherichia coli* in faecal droplets from bats and poultry in Nigeria. Vet. Ital.

[73] Aladejana O.M, Oluduro A.O, Ogunlade A.O, Famurewa O (2022). Molecular characterization of resistance and virulence genes in *Escherichia coli* isolated from bats (*Eidolon helvum*) Faeces in Osun State, Nigeria. J. Adv. Microbiol.

[74] Nguema P.P.M, Onanga R, Atome G.R.N, Mbeang J.C.O, Mabika A.M, Yaro M, Lounnas M, Dumont Y, Zohra Z.F, Godreuil S, Bretagnolle F (2020). Characterization of ESBL-producing enterobacteria from fruit bats in an unprotected area of Makokou, Gabon. Microorganisms.

[75] Oladiran F, Oluwayemisi O.A, Abike O, Olufunke A, Modupe O (2022). Molecular characterization of resistance and virulence genes in *Escherichia coli* isolated from bats (*Eidolon helvum*) Faeces in Osun State, Nigeria. J. Adv. Microbiol.

[76] McDougall F.K, Boardman W.S, Power M.L (2021). Characterization of beta-lactam-resistant *Escherichia coli* from Australian fruit bats indicates anthropogenic origins. Microb. Genom.

[77] McDougall F, Boardman W, Power M (2022). High prevalence of beta-lactam-resistant *Escherichia coli* in South Australian grey-headed flying fox pups (*Pteropus poliocephalus*). Microorganisms.

[78] Gauba A, Rahman K.M (2023). Evaluation of antibiotic resistance mechanisms in Gram-negative bacteria. Antibiotics (Basel).

[79] Lungu B.C, Hutu I, Barrow P.A (2023). Molecular characterisation of antimicrobial resistance in *E. coli* isolates from piglets in the West Region of Romania. Antibiotics (*Basel*).

[80] Ribeiro J, Silva V, Monteiro A, Vieira-Pinto M, Igrejas G, Reis F.S, Barros L, Poeta P (2023). Antibiotic resistance among gastrointestinal bacteria in broilers:A review focused on *Enterococcus* spp. and *Escherichia coli*. Animals (*Basel*).

[81] Lagerstrom K.M, Hadly E.A (2021). The under-investigated wild side of *Escherichia coli*:Genetic diversity, pathogenicity and antimicrobial resistance in wild animals. Proc. R. Soc. B.

[82] Almansour A.M, Alhadlaq M.A, Alzahrani K.O, Mukhtar L.E, Alharbi A.L, Alajel S.M (2023). The silent threat:Antimicrobial-resistant pathogens in food-producing animals and their impact on public health. Microorganisms.

[83] Casey J.A, Tartof S.Y, Davis M.F, Nachman K.E, Price L, Liu C, Yu K, Gupta V, Innes G.K, Tseng H.F, Do V, Pressman A.R, Rudolph K.E (2023). Impact of a statewide livestock antibiotic use policy on resistance in human urine *Escherichia coli* isolates:A synthetic control analysis. Environ. Health Perspect.

[84] Grudlewska-Buda K, Bauza-Kaszewska J, Wiktorczyk-Kapischke N, Budzyńska A, Gospodarek-Komkowska E, Skowron K (2023). Antibiotic resistance in selected emerging bacterial foodborne pathogens-an issue of concern?. *Antibiotics* (Basel).

[85] Tiedje J.M, Fu Y, Mei Z, Schäffer A, Dou Q, Amelung W, Elsner M, Adu-Gyamfi J, Heng L, Virta M, Jiang X, Smidt H, Topp E, Wang F (2023). Antibiotic resistance genes in food production systems support one health opinions. Curr. Opin. Environ. Sci. Health.

[86] Collineau L, Bourély C, Rousset L, Berger-Carbonne A, Ploy M.C, Pulcini C, Colomb-Cotinat M (2023). Towards one health surveillance of antibiotic resistance:Characterisation and mapping of existing programmes in humans, animals, food and the environment in France, 2021. Euro Surveill.

[87] Wibisono F.J, Sumiarto B, Untari T.R.I, Effendi M.H, Permatasari D.A, Witaningrum A.M (2020). Pattern of antibiotic resistance on extended-spectrum beta-lactamases genes producing *Escherichia coli* on laying hens in Blitar, Indonesia. Biodiversitas.

[88] Faridah H.D, Dewi E.K, Effendi M.H, Plumeriastuti H (2020). A review of antimicrobial resistance (AMR) of *Escherichia coli* on livestock and animal products:Public health importance. Syst. Rev. Pharm.

[89] Islam M.S, Hossain M.J, Sobur M.A, Punom S.A, Rahman A.M.M.T, Rahman M.T (2023). A systematic review on the occurrence of antimicrobial-resistant *Escherichia coli* in poultry and poultry environments in Bangladesh between 2010 and 2021. Biomed. Res. Int.

[90] Ugbo E.N, Jacob J.I, Effendi M.H, Witaningrum A.M, Agumah B.N, Ugbo A.I, Moses B.I (2023). Poultry slaughterhouse wastewater as reservoirs for spreading extended-spectrum beta-lactamase-producing *Escherichia coli* in Abakaliki, Nigeria. Biodiversitas.

[91] Williams A.D, Rousham E, Neal A.L, Amin M.B, Hobman J.L, Stekel D, Islam M.A (2023). Impact of contrasting poultry exposures on human, poultry, and wastewater antibiotic resistomes in Bangladesh. Microbiol. Spectr.

[92] Yanestria S.M, Dameanti F.N.A.E.P, Musayannah B.G, Pratama J.W.A, Witaningrum A.M, Effendi M.H, Ugbo E.N (2022). Antibiotic resistance pattern of Extended-Spectrum -Lactamase (ESBL) producing *Escherichia coli* isolated from broiler farm environment in Pasuruan district, Indonesia. Biodiversitas.

[93] Okeke I.N, Fayinka S.T, Lamikanra A (2000). Antibiotic resistance in *Escherichia coli* from Nigerian students, 1986–1998. Emerg. Infect. Dis.

[94] Nowak K, Fahr J, Weber N, Lübke-Becker A, Semmler T, Weiss S, Mombouli J.V, Wieler L.H, Guenther S, Leendertz F.H, Ewers C (2017). Highly diverse and antimicrobial susceptible *Escherichia coli* display a naïve bacterial population in fruit bats from the Republic of Congo. PLoS One.

[95] Li B, Qiu Y, Song Y, Lin H, Yin H (2019). Dissecting horizontal and vertical gene transfer of antibiotic resistance plasmid in bacterial community using microfluidics. Environ. Int.

[96] Karaiskos I, Giamarellou H (2020). Carbapenem-sparing strategies for ESBL producers:When and how. Antibiotics (*Basel*).

[97] Larramendy S, Deglaire V, Dusollier P, Fournier J.P, Caillon J, Beaudeau F, Moret L (2020). Risk factors of extended-spectrum beta-lactamases-producing *Escherichia coli* community acquired urinary tract infections:A systematic review. Infect. Drug Resist.

[98] Widodo A, Effendi M.H, Khairullah A.R (2020). Extended-spectrum beta-lactamase (ESBL)-producing *Escherichia coli* from livestock. Sys. Rev. Pharm.

[99] Tseng C.H, Liu C.W, Liu P.Y (2023). Extended-spectrum -lactamases (ESBL) producing bacteria in animals. Antibiotics (Basel).

[100] Gorbunova V, Seluanov A, Kennedy B.K (2020). The world goes bats:Living longer and tolerating viruses. Cell Metab.

[101] Wilkinson G.S, Adams D.M (2019). Recurrent evolution of extreme longevity in bats. Biol. Lett.

[102] Devnath P, Karah N, Graham J.P, Rose E.S, Asaduzzaman M (2022). Evidence of antimicrobial resistance in bats and its planetary health impact for surveillance of zoonotic spillover events:A scoping review. Int. J. Environ. Res. Public Health.

[103] Santosa S, Soekendarsi E, Hasan M.S, Fahruddin F, Priosambodo D (2020). Potential of community based ecotourism of bats population (Megachiroptera) in Soppeng Regency, Indonesia. Int. J. Appl. Biol.

[104] Stidsholt L, Scholz C, Hermanns U, Teige T, Post M, Stapelfeldt B, Reusch C, Voigt C.C (2024). Low foraging rates drive large insectivorous bats away from urban areas. Glob. Chang. Biol.

[105] Chan A.A.Q, Aziz S.A, Clare E.L, Coleman J.L (2021). Diet, ecological role and potential ecosystem services of the fruit bat, *Cynopterus brachyotis*, in a tropical city. Urban Ecosyst.

[106] Zhang M, Wei X (2021). Deforestation, forestation, and water supply. Science.

[107] Ajuzieogu C, Nwankwo U.G, Ikedianya N (2024). Impact of bat guano fertilizer on soil bacteria community structure and antibiogram of associated bacteria:An alert to food insecurity. Asian J. Res. Biosci.

[108] Nappier S.P, Liguori K, Ichida A.M, Stewart J.R, Jones K.R (2020). Antibiotic resistance in recreational waters:State of the science. Int. J. Environ. Res. Public Health.

[109] Ramos D.A, Pulgarín J.A.H, Gómez G.A.M, Alzate J.A, Gómez J.C.O, Bonilla I.C, Mosquera C.V (2020). Geographic mapping of Enterobacteriaceae with extended-spectrum -lactamase (ESBL) phenotype in Pereira, Colombia. BMC Infect. Dis.

[110] Perestrelo S, Carreira G.C, Valentin L, Fischer J, Pfeifer Y, Werner G, Schmiedel J, Falgenhauer L, Imirzalioglu C, Chakraborty T, Käsbohrer A (2022). Comparison of approaches for source attribution of ESBL-producing *Escherichia coli* in Germany. PLoS One.

[111] Hayer J, Salgado-Caxito M, Opazo-Capurro A, Muñoz P.G, Millán J, Piñeiro A, Munita J.M, Rivas L, Benavides J.A (2023). Multiple clonal transmissions of clinically relevant extended-spectrum beta-lactamase-producing *Escherichia coli* among livestock, dogs, and wildlife in Chile. J. Glob. Antimicrob. Resist.

[112] Kulik K, Lenart-Boroń A, Wyrzykowska K (2023). Impact of antibiotic pollution on the bacterial population within surface water with special focus on mountain rivers. Water.

[113] Sabença C, Romero-Rivera M, Barbero-Herranz R, Sargo R, Sousa L, Silva F, Lopes F, Abrantes A.C, Vieira-Pinto M, Torres C, Igrejas G, Del Campo R, Poeta P (2024). Molecular characterization of multidrug-resistant *Escherichia coli* from fecal samples of wild animals. Vet. Sci.

[114] Agustin A.L.D, Effendi M.H, Tyasningsih W, Khairullah A.R (2024). Cave bats as carriers of extended spectrum beta-lactamase produced by *Escherichia coli* from the Island of Lombok, Indonesia. Indian J. Anim. Res.

[115] Palmeira J.D, Haenni M, Madec J.Y, Ferreira H.M.N (2021). First global report of plasmid-mediated mcr-1 and extended-spectrum beta-lactamase-producing *Escherichia coli* from sheep in Portugal. Antibiotics (*Basel*).

[116] Oliveira R.P, da Silva J.S, da Silva G.C, Rosa J.N, Bazzolli D.M.S, Mantovani H.C (2024). Prevalence and characteristics of ESBL-producing *Escherichia coli* in clinically healthy pigs:Implications for antibiotic resistance spread in livestock. J. Appl. Microbiol.

[117] Belmahdi M, Chenouf N.S, Belkacem A.A, Martinez-Alvarez S, Pino-Hurtado M.S, Benkhechiba Z, Lahrech S, Hakem A, Torres C (2022). Extended spectrum -Lactamase-producing *Escherichia coli* from poultry and wild birds (Sparrow) in Djelfa (Algeria), with frequent detection of CTX-M-14 in sparrow. Antibiotics (*Basel*).

[118] Clemente L, Leão C, Moura L, Albuquerque T, Amaro A (2021). Prevalence and characterization of ESBL/AmpC producing *Escherichia coli* from Fresh meat in Portugal. Antibiotics (*Basel*).

[119] Carvalho I, Carvalho J.A, Martínez-Álvarez S, Sadi M, Capita R, Alonso-Calleja C, Rabbi F, Dapkevicius M.L.N.E, Igrejas G, Torres C, Poeta P (2021). Characterization of ESBL-producing *Escherichia coli* and *Klebsiella pneumoniae* isolated from clinical samples in a Northern Portuguese hospital:Predominance of CTX-M-15 and high genetic diversity. Microorganisms.

[120] Benavides J.A, Salgado-Caxito M, Opazo-Capurro A, Muñoz P.G, Piñeiro A, Medina M.O, Rivas L, Munita J, Millán J (2021). ESBL-producing *Escherichia coli* carrying CTX-M genes circulating among livestock, dogs, and wild mammals in small-scale farms of central chile. Antibiotics (*Basel*).

[121] Bazalar-Gonzales J, Silvestre-Espejo T, Cueva C.R, Huamán D.C, León Y.I, Espinoza L.L, Alcántara R.R, Hernández L.M (2024). Genomic insights into ESBL-producing *Escherichia coli* isolated from non-human primates in the Peruvian Amazon. Front. Vet. Sci.

[122] Cortez-Sandoval V, González R, Ramos D (2022). Detection of extended-spectrum -lactamase-producing Enterobacteriaceae (ESBL) isolated from retail chicken meat in a district of Lima, Peru. Rev. Investig. Vet. Perú.

[123] Effendi M.H, Wibisono F.J, Witaningrum A.M, Permatasari D.A (2021). Identification of *bla*TEM and *bla*SHV Genes of extended spectrum beta lactamase (ESBL) producing *Escherichia coli* from broiler chickens in Blitar, Indonesia. Syst. Rev. Pharm.

[124] Hardiati A, Safika S, Wibawan I.W.T, Indrawati A, Pasaribu F.H (2021). Isolation and detection of antibiotic resistance genes of *Escherichia coli* from broiler farms in Sukabumi, Indonesia. J. Adv. Vet. Anim. Res.

[125] Ansharieta R, Ramandinianto S.C, Effendi M.H, Plumeriastuti H (2021). Molecular identification of *bla*CTX-M and *bla*TEM genes encoding extended-spectrum -lactamase (ESBL) producing *Escherichia coli* isolated from raw cow's milk in East Java, Indonesia. Biodiversitas.

[126] Candra I.K.B, Yanto F, Suranadi I.W, Fatmawati N.N.D (2021). Characteristic of extended spectrum -lactamase-producing Enterobacteriaceae from fecal carriage isolates of intensive care unit patients at Sanglah Hospital, Bali, Indonesia. Open Microbiol. J.

[127] Odumosu B.T, Obeten H.I, Bamidele T.A (2021). Incidence of multidrug-resistant *Escherichia coli* harbouring *bla*TEM and *tet*A genes isolated from seafoods in Lagos Nigeria. Curr. Microbiol.

[128] Anueyiagu K.N, Agusi E.R, Audu B.J, Achi L.C (2022). Phylodiversity of *bla*TEM and *bla*CTX-M genes of coliform isolates from ruminant mastitis in Plateau State, Nigeria. J. Anim. Sci. Vet. Med.

[129] Fashae K, Engelmann I, Monecke S, Braun S.D, Ehricht R (2021). Molecular characterisation of extended-spectrum -lactamase producing *Escherichia coli* in wild birds and cattle, Ibadan, Nigeria. BMC Vet. Res.

[130] Aworh M.K, Ekeng E, Nilsson P, Egyir B, Owusu-Nyantakyi C, Hendriksen R.S (2022). Extended-spectrum -lactamase-producing *Escherichia coli* among humans, beef cattle, and abattoir environments in Nigeria. Front. Cell. Infect. Microbiol.

[131] Widodo A, Lamid M, Effendi M.H, Tyasningsih W, Raharjo D, Khairullah A.R, Kurniawan S.C, Yustinasari L.R, Riwu K.H.P, Silaen O.S.M (2023). Molecular identification of *bla*TEM and *bla*CTX-M genes in multidrug-resistant *Escherichia coli* found in milk samples from dairy cattle farms in Tulungagung, Indonesia. J. Vet. Res.

[132] Ramos S, Silva V, Dapkevicius M.D.L.E, Caniça M, Tejedor-Junco M.T, Igrejas G, Poeta P (2020). *Escherichia coli* as commensal and pathogenic bacteria among food-producing animals:Health implications of extended spectrum -lactamase (ESBL) production. Animals (*Basel*).

[133] Da Silva S.K.S.M, Fuentes-Castillo D.A, Ewbank A.C, Sacristán C, Catão-Dias J.L, Sevá A.P, Lincopan N, Deem S.L, Feitosa L.C.S, Catenacci L.S (2024). ESBL-producing enterobacterales at the human-domestic animal-wildlife interface:A one health approach to antimicrobial resistance in Piauí, Northeastern Brazil. Vet. Sci.

[134] Founou R.C, Founou L.L, Essack S.Y (2017). Clinical and economic impact of antibiotic resistance in developing countries:A systematic review and meta-analysis. PLoS One.

[135] Collingwood J.D, Wang L, Aban I.B, Yarbrough A.H, Boppana S.B, Dangle P.P (2023). Risk factors for community acquired pediatric urinary tract infection with extended-spectrum- -lactamase Escherichia coli - A case-control study. J. Pediatr. Urol.

[136] El-Mokhtar M, Daef E, Hussein A.A.R.M, Hashem M.K, Hassan H.M (2021). Emergence of nosocomial pneumonia caused by colistin-resistant *Escherichia coli* in patients admitted to chest intensive care unit. Antibiotics (*Basel*).

[137] Palacios-Zertuche J.T, Limas-Rodriguez Q.G, Gonzalez-Cantu C.M, Perez-Salazar D.A, Saldivar-Martinez D.E, Munoz-Maldonado G.E (2016). Case report:Intestinal tuberculosis with perforation of the colon and psoas abscess associated with *Escherichia coli* ESBL. Med. Univ.

[138] Lubwama M, Kateete D.P, Katende G, Kigozi E, Orem J, Phipps W, Bwanga F (2024). CTX-M, TEM, and SHV genes in *Escherichia coli, Klebsiella pneumoniae,* and *Enterobacter spp* isolated from hematologic cancer patients with bacteremia in Uganda. Infect. Drug Resist.

[139] Bezabih Y.M, Sabiiti W, Alamneh E, Bezabih A, Peterson G.M, Bezabhe W.M, &Roujeinikova A (2021). The global prevalence and trend of human intestinal carriage of ESBL-producing *Escherichia coli* in the community. Journal of Antimicrobial Chemotherapy.

[140] Kasanga M, Kwenda G, Wu J, Kasanga M, Mwikisa M.J, Chanda R, Mupila Z, Yankonde B, Sikazwe M, Mwila E, Shempela D.M, Solochi B.B, Phiri C, Mudenda S, Chanda D (2023). Antimicrobial resistance patterns and risk factors associated with ESBL-producing and MDR *Escherichia coli* in hospital and environmental settings in Lusaka, Zambia:Implications for one health, antimicrobial stewardship and surveillance systems. Microorganisms.

[141] Kassem I.I, Nasser N.A, Salibi J (2020). Prevalence and loads of fecal pollution indicators and the antibiotic resistance phenotypes of *Escherichia coli* in raw minced beef in Lebanon. Foods.

[142] Schaufler K, Semmler T, Wieler L.H, Trott D.J, Pitout J, Peirano G, Bonnedahl J, Dolejska M, Literak I, Fuchs S, Ahmed N, Grobbel M, Torres C, McNally A, Pickard D, Ewers C, Croucher N.J, Corander J, Guenther S (2019). Genomic and functional analysis of emerging virulent and multidrug-resistant *Escherichia coli* lineage sequence TYPE 648. Antimicrob. Agents Chemother.

[143] Mandujano-Hernández A, Martínez-Vázquez A.V, Paz-González A.D, Herrera-Mayorga V, Sánchez-Sánchez M, Lara-Ramírez E.E, Vázquez K, de Jesús de Luna-Santillana E, Bocanegra-García V, Rivera G (2024). The global rise of ESBL-producing *Escherichia coli* in the livestock sector:A five-year overview. Animals (*Basel*).

[144] Perestrelo S, Amaro A, Brouwer M. S, Clemente L, Ribeiro Duarte A. S, Kaesbohrer A, Karpíšková R, Lopez-Chavarrias V, Morris D, Prendergast D, Pista A, Silveira L, Skarżyńska M, Slowey R, Veldman K.T, Zając M, Burgess C, Alvarez J (2023). Building an international one health strain level database to characterise the epidemiology of AMR threats:ESBL-AmpC producing *E. coli* as an example-challenges and perspectives. Antibiotics (*Basel*).

[145] Lai C.C, Lee K, Xiao Y, Ahmad N, Veeraraghavan B, Thamlikitkul V, Tambyah P.A, Nelwan R.H, Shibl A.M, Wu J.J, Seto W.H, Hsueh P.R (2014). High burden of antimicrobial drug resistance in Asia. J. Glob. Antimicrob. Resist.

[146] Furusawa M, Widgren S, Evers E.G, Fischer E.A (2024). Quantifying health risks from ESBL-producing *Escherichia coli* in Dutch broiler production chains and potential interventions using compartmental models. Prev. Vet. Med.

[147] Okada N, Takahashi M, Yano Y, Sato M, Abe A, Ishizawa K, Azuma M (2022). Hospital outbreak of extended-spectrum beta-lactamase-producing *Escherichia coli* potentially caused by toilet and bath chair use. Infect. Prev. Pract.

[148] Kajihara T, Yahara K, Stelling J, Eremin S.R, Tornimbene B, Thamlikitkul V, Hirabayashi A, Anzai E, Wakai S, Matsunaga N, Hayakawa K, Ohmagari N, Sugai M, Shibayama K (2020). Comparison of de-duplication methods used by WHO Global Antimicrobial Resistance Surveillance System (GLASS) and Japan Nosocomial Infections Surveillance (JANIS) in the surveillance of antimicrobial resistance. PLoS One.

[149] Dimande P, Arrobas M, Rodrigues M.Â (2023). Under a tropical climate and in sandy soils, bat guano mineralises very quickly, behaving more like a mineral fertiliser than a conventional farmyard manure. Agronomy.

[150] Murugaiyan J, Kumar P.A, Rao G.S, Iskandar K, Hawser S, Hays J.P, Mohsen Y, Adukkadukkam S, Awuah W.A, Jose R.A.M, Sylvia N, Nansubuga E.P, Tilocca B, Roncada P, Roson-Calero N, Moreno-Morales J, Amin R, Kumar B.K, Kumar A, Toufik A.R, Zaw T.N, Akinwotu O.O, Satyaseela M.P, van Dongen M.B.M (2022). Progress in alternative strategies to combat antimicrobial resistance:Focus on antibiotics. Antibiotics (*Basel*).

[151] Widodo A, Lamid M, Effendi M.H, Khairullah A.R, Riwu K.H.P, Yustinasari L.R, Kurniawan S.C, Ansori A.N.M, Silaen O.S.M, Dameanti F.N.A.E.P (2022). Antibiotic sensitivity profile of multidrug-resistant (MDR) *Escherichia coli* isolated from dairy cow's milk in Probolinggo, Indonesia. Biodiversitas.

[152] Pradika A.Y, Chusniati S, Purnama M.T.E, Effendi M.H, Yudhana A, Wibawati P.A (2019). Total test of *Escherichia coli* on fresh cow milk at dairy farmer cooperative (KPSP) Karyo Ngremboko Purwoharjo Banyuwangi. J. Med. Vet.

